# Imine-Linked Polymer Based Nitrogen-Doped Porous Activated Carbon for Efficient and Selective CO_2_ Capture

**DOI:** 10.1038/srep38614

**Published:** 2016-12-13

**Authors:** Akram Alabadi, Hayder A. Abbood, Qingyin Li, Ni Jing, Bien Tan

**Affiliations:** 1 School of Chemistry and Chemical Engineering, Huazhong University of Science and Technology, Luoyu Road No. 1037, Wuhan, 430074, China; 2 South Refineries Company, Ministry of Oil, Basra, 61006, Iraq; 3 Material Engineering Department, College of Engineering, University of Basrah, Basarah, 61006, Iraq

## Abstract

The preparation of nitrogen-doped activated carbon (NACs) has received significant attention because of their applications in CO_2_ capture and sequestration (CCS) owing to abundant nitrogen atoms on their surface and controllable pore structures by carefully controlled carbonization. We report high-surface-area porous N-doped activated carbons (NAC) by using soft-template-assisted self-assembly followed by thermal decomposition and KOH activation. The activation process was carried out under different temperature conditions (600–800 °C) using polyimine as precursor. The NAC-800 was found to have a high specific surface area (1900 m^2^ g^−1^), a desirable micropore size below 1 nm and, more importantly, a large micropore volume (0.98 cm^3^ g^−1^). NAC-800 also exhibits a significant capacity of CO_2_ capture i.e., over 6. 25 and 4.87 mmol g^−1^ at 273 K and 298 K respectively at 1.13 bar, which is one of among the highest values reported for porous carbons so far. Moreover, NAC also shows an excellent separation selectivity for CO_2_ over N_2_.

The increasing level of atmospheric CO_2_ is great concern due to its impact on global warming and climate change. Serious efforts are, therefore, being made not only to increase the public awareness but also to develop strategies to minimize the emission of flue gases from the power plants utilizing fossil fuels and resources. To minimize the impact on the environment, the anthropogenic CO_2_ emission after combustion of flue gas must be decreased^1^. To achieve this, the highly selective CO_2_ adsorbing materials are being developed/explored and carbon capture and storage (CCS) materials are among the attractive materials in this regard to reduce CO_2_ concentration from the atmosphere because of their lower transportation and storage cost compared to the CO_2_ capture cost^2^,^3^,^4^.

Over the past decades, a variety of solid-based microporous materials have been used for CO_2_ capture and storage such as metal organic frameworks (MOFs), hyper-crosslinking polymer (HCPs), covalent organic frameworks (COFs), activated carbons (ACs), functionalized graphene, chemically modified mesoporous materials, and so forth^5^,^6^. Among these, activated carbons, due to their recyclability, availability, easier synthesis and strong adsorption with large surface areas, have found their widespread applications as microporous materials for carbon dioxide capture and storage or separation by physical adsorption^7^. Microporous materials including activated carbons (ACs) have been regarded as competent candidates for carbon dioxide capture and storage at ambient temperatures, due to their large surface areas, high physical and chemical stability and low density^8^. To enhance the uptake capacity of activated carbons for carbon dioxide at ambient temperatures, researchers have increased the surface area and regulated the appropriate pore size. Presently, some researchers have even tried to increase the adsorption capacity for carbon dioxide by creating special active sites such as heteroatoms and different organic groups into the activated carbons to improve the interactions between the adsorbate and the functional groups in porous carbons^9^,^10^,^11^.

Hydrothermal carbonization (HTC) is a good way to obtain useful carbon materials and to prepare functionally diverse carbon materials so far^12^, both physical and chemical activation techniques have become well-established for the preparation of activated carbons. In the physical activation, the precursor materials are carbonized at high temperatures with a flow of gases (CO_2_ or steam), while in chemical activation the precursors are first impregnated or physically mixed with chemical agents and then heated at the target temperature under inert atmosphere. To improve the porosity of the activated carbons, many activation parameters are to be taken into consideration, such as carbonization temperature, the type of activation agent, the weight ratio between activation agents and the sample, and the time of carbonization held at the target temperature^11^,^13^ etc., all of which have their own significance in the development of porosity of activated carbons. But it is still unclear which factor has the largest effect on controlling the porosity in activated carbons.

Many types of synthetic polymers have been selected as the carbon source since they contain amine groups such as poly acrylonitrile (PAN)^14^, melamine formaldehyde resin^15^, polypyrrole^16^ and poly aniline^17^. Nitrogen has become one of the most widely studied heteroatoms in the optimization of carbonaceous materials used as supercapacitors and gas adsorption and storage^18^. Nitrogen-containing carbonaceous materials are prepared by two primary methods: the post treatment and *in situ* method. In the former one, carbonaceous materials are treated with nitrogen containing compounds such as ammonia^19^, urea and melamine^20^ to incorporate nitrogen functional groups. It is commonly used to produce various porous carbons, such as carbon nanotubes, graphene, and activated carbons. In the latter one, many nitrogen-containing precursors are directly used as precursors to produce nitrogen-functionalized carbonaceous materials^21^, including poly aniline (PNI), poly pyrrole (PPy)^22^, melamine analogues^23^ and poly acrylonitrile^14^. However, CO_2_ adsorption properties of N-doped porous materials have been rarely investigated so far due to its Lewis-acidic and quadrupolar momentum^24^. Kaskel *et al*. investigated porous polyimine as the precursor^18^. Sun *et al*. synthesized hierarchical macroporous N-doped carbons (MCN) with ordered three-dimensional periodic structures^25^. However, CO_2_ is expected to interact robustly with high polar imines functionalities in the polymer^26^. Gas adsorption and desorption of porous materials depend not only on the high specific surface area, micropore volume or pore size but also on the chemical composition and structural features of the materials^27^. To create porous carbons with high porosity and a pore size to facilitate diffusion and adsorption/desorption of gas molecules, most of the synthesis is achieved by procedures using an approximate template which is quite intricate and time consuming or using activation, which is considered to be easier and cheaper compared with the template method^28^. However, it is still a major challenge to explore a simple synthetic procedure to produce highly efficient porous materials for CO_2_ capture and storage.

Here, we present a facile strategy, which involves a new type of polyimine direct pyrolysis with KOH as a chemical activating agent in the tubular furnace by adjusting the activation temperature, to prepare (i) highly ordered N-doped porous activated carbon using polyimine as an organic precursor (ii) naturally occurring nitrogen is preserved as dopants to motivate molecular adsorption, *e.g.* for CO_2_ capture. The resultant N-doped porous carbon exhibits a high CO_2_ adsorption capacity, and more interestingly a high selectivity for CO_2_ over N_2_.

## Result and Discussion

### Morphology of sample

**Figure 1:**
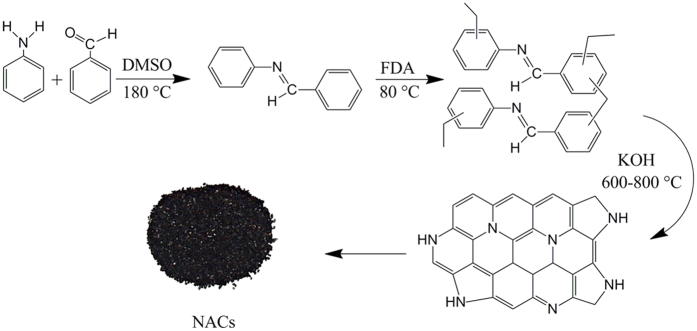
Representative schematic diagram of NACs synthesis.

The overall process for the synthesis of nitrogen- doped porous activated carbons (NACs) is shown in Fig. 1. In the first step, imine-linked monomer was prepared by the reaction of aniline and benzaldehyde in the presence of DMSO as a solvent. Then, hyper crosslinked polymer was synthesized by *Friedel-Crafts* reaction to produce polyimine as the carbon precursor. NACs samples were prepared from polyimine carbonization in the range temperature range (600–800 °C).

**Figure 2:**
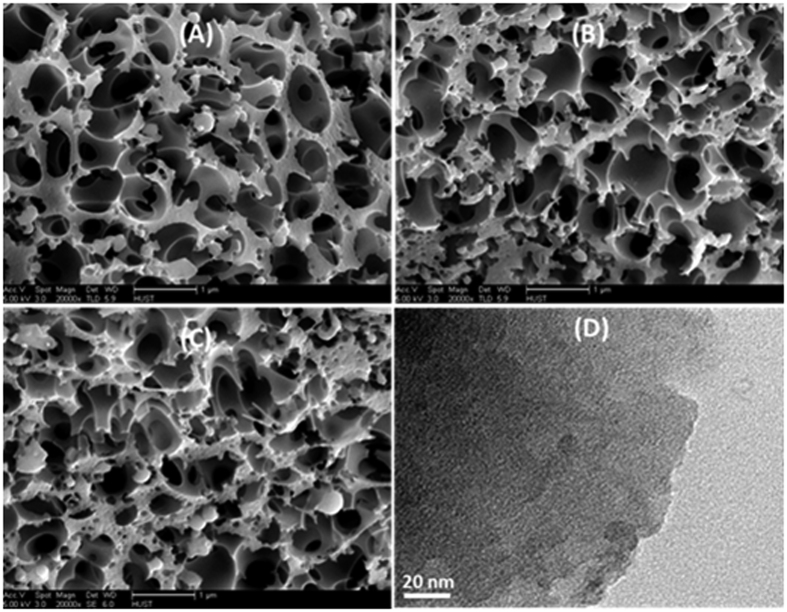
SEM images of (**A**) NAC-600 (**B**) NAC-700 (**C**) NAC-800 and TEM image of (**D**) NAC-800.

The morphological features of the NACs were characterized by FE-SEM and HRTEM. The scanning electron microscope (SEM) images exhibit an increased porosity with an increase in the pyrolysis temperature. NACs consist of a lot of cavities and relatively uniform pores and highly interconnected sub-voids, indicating generation of porosity after the reaction of the alkaline reagent KOH during activation as is shown in Fig. 2A–C. The high-resolution transmission electron microscope (HRTEM) was employed to further examine the morphology and textural features of as-synthesized microporous NACs. As evident from Fig. 2(D), bright contrast points on the under focused images represent the pore location, whereas dark contrast cores display empty cavities. It is obvious, however, that they NACs have highly disordered and dense pores.

**Figure 3:**
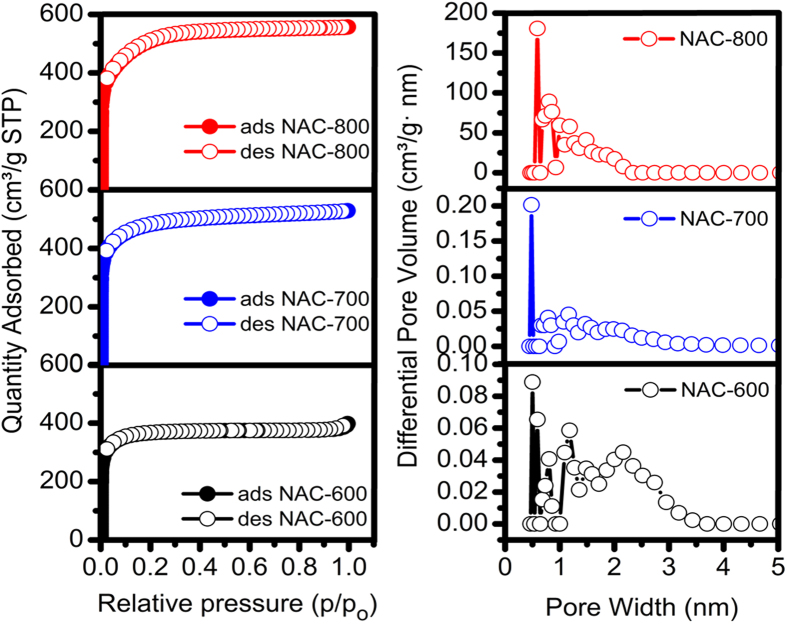
Nitrogen adsorption (solid symbols) and desorption (open symbols) isotherms and pore size distributions of NACs, analyzed on N_2_ adsorption isotherms measured at 77 K using the NLDFT model.

Nitrogen adsorption/desorption isotherms of the N-ACs are presented in Fig. 3. All isotherms quite closely correspond to the Type I adsorption according to IUPAC classification. Rapid N_2_ uptake at relatively low pressures (P/P_0_ < 0.05) can be observed, which is typical characteristic of microporous materials and there is no tangible hysteresis loop between adsorption and desorption isotherm for these carbons, which is an indicative of their ultra-microporous nature. BET surface area and micropore volume values calculated from relative pressure between 0.01 and 0.1 are listed on Table 1. NAC-800 shows the highest BET surface area of 1900 m^2^ g^−1^ with a total pore volume (Vt) up to 0.98 cm^3^ g^−1^, while the BET surface area, the total pore volume for NAC-700 (1719 m^2^ g^−1^, 0.85 cm^3^ g^−1^) and NAC-600 (1681 m^2^ g^−1^, 0.80 cm^3^ g^−1^) indicate that the heat treatment at elevated temperatures in the presence of KOH imparts surface roughness to the carbon material and leads to an increase in surface area and porosity. For chemical activation, the specific surface area, the total pore volume and the micropore volume of the carbon samples increase remarkably at higher activation temperature (800 °C). The pore size distributions are shown in Fig. 3. The pores are exclusively microporous with less mesoporous (pores, 2 nm) and the highly activated sample NAC-800 reveals a small amount of mesopores up to 2 nm. Moreover, the most of the pores are less than 2 nm in all samples indicating the pore development at both micro- and meso-pore ranges. NAC-800 possesses a relatively high volume of micropores, while NAC-700 and NAC-600 shows a marked decrease in the micropore volume.

**Figure 4:**
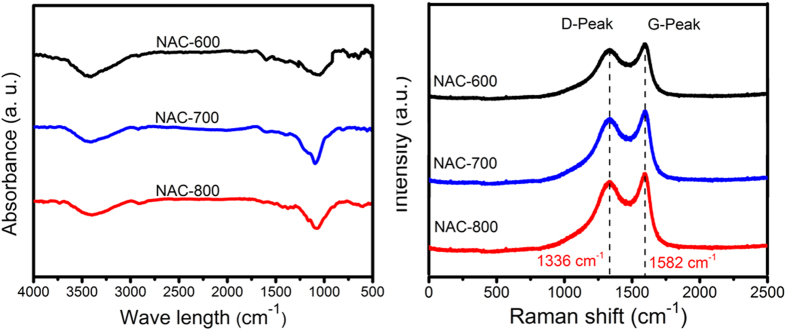
FTIR spectra (left) and Raman spectroscopy (right) showing characteristic D and G peak of NACs samples.

The FTIR spectra (Fig. 4 left) of NACs show that the characteristic broad band at ca 3417 cm^−1^ can be assigned to N-H and/or O-H stretching vibration. The weak band at 2925 cm^−1^ corresponds to C-H bond, and the weak band of 1627 cm^−1^ is attributed to the distinctive absorbance of C-H bonds of benzene rings as well as the C=N bonds from the carbon framework. The peaks at 1160 cm^−1^ suggest the presence of the C-N stretching vibration. The broad peak of 1100 cm^−1^ is associated with C-N stretching vibration. The FTIR analysis, therefore, confirms the existence of N-H and C-N species in the carbon samples. The elemental analyses show the heteroatom- doped characteristics of NACs, which consist mainly of C, N and O. It is also obvious that there is an increase in the element of C, while N and O content gradually decreased with increasing carbonization temperatures. Nitrogen is present in all NAC samples ranging typically from 1.73% to 2.16%, which suggests the presence of thermally stable nitrogen in the NAC samples during carbonization and activation.

To further understand the chemistry of NACs, their Raman spectrum and the corresponding fitted data are shown in Fig. 4 right. The spectrum shows two peaks, one peak (G-band) at ∼1582 cm^−1^ due to the E_2_g phonon of sp^2^ carbon atoms, characteristic of features of the graphitic layers and corresponds to the tangential vibration of the carbon atoms and the other (D-band) is located at ∼1336 cm^−1^, which can be attributed to A_1_g symmetry, corresponding to the disordered carbon or defective graphite structures. The intensity ratio of “D/G”, also called the “R-value”, reflects the range of structurally ordered graphite in the porous carbon materials and reveals the graphitization degree of the carbonaceous materials. Notably, the intensity of D band is higher than that of G band due to the defects and partially disordered structure of the samples. It is worth noting that the intensity ratio (D/G) of as-synthesized samples decreases with increasing temperature. Therefore, the intensity ratio of NAC-800 is lower compared to that of NAC-600 and NAC-700.

**Figure 5:**
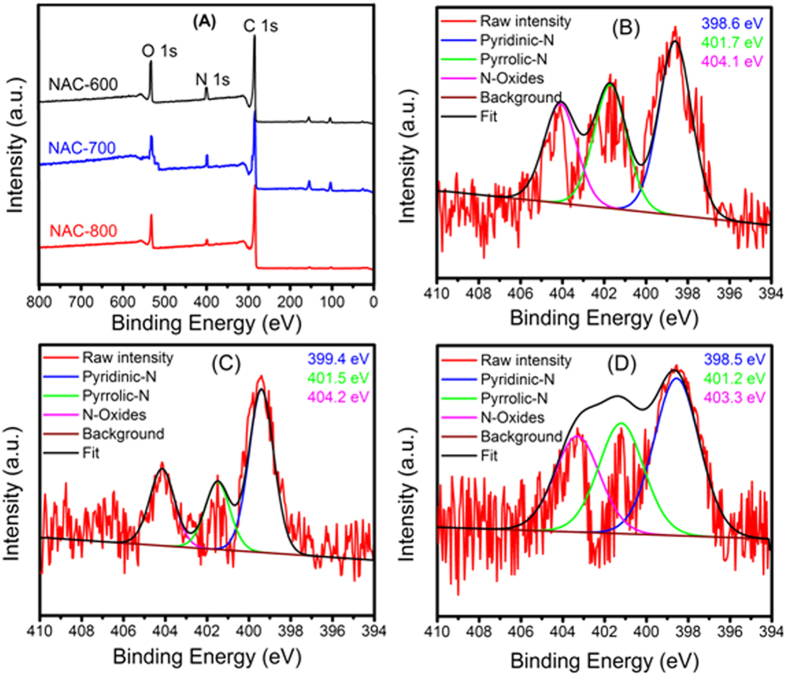
XPS spectra of NACs (**A**) survey of NAC-800 and N1s XPS spectra of NAC-600 (**B**), NAC-700 (**C**) NAC-800 (**D**).

In order to obtain further information about the elemental composition and nitrogen-carbon bonding configurations formed on the surface of the NAC samples, X-ray photoelectron spectroscopy (XPS) analysis was employed to quantitatively analyze the nitrogen-doping. XPS spectrum displays several peaks at 284 eV, 399 eV and 532 eV, which can be attributed to C, N, and O respectively as shown in Fig. 5A. For all samples, the XPS N 1 s core signal is de-convoluted into three peaks with binding energies at range 398.6–399.4 eV assigned to the pyridinic-N (N-6), with the nitrogen atom in a six-membered ring and contributing with one p-electron to the aromatic p-system. The pre-dominant peaks are situated between 401.2 eV and 401.7 eV, which is assigned to pyrrolic-N (N-5), with two p-electrons contributing to the p-system. The third signal around 403.3–404.2 eV corresponds to a classic graphitic-type N-oxides (Fig. 5B–D). The weight percentages of C, O, and N in the samples are summarized in Table 1. The surface nitrogen content is obtained from XPS measurement in a range 1.53–2.31 wt%. These values are comparable with the CHN results, indicating the homogeneous dispersion of nitrogen species in the carbon matrix. However, high temperature thermal treatments (carbonization and activation) result in the increase in the C ratio and decrease in N ratio.

### CO_2_ adsorption

**Figure 6:**
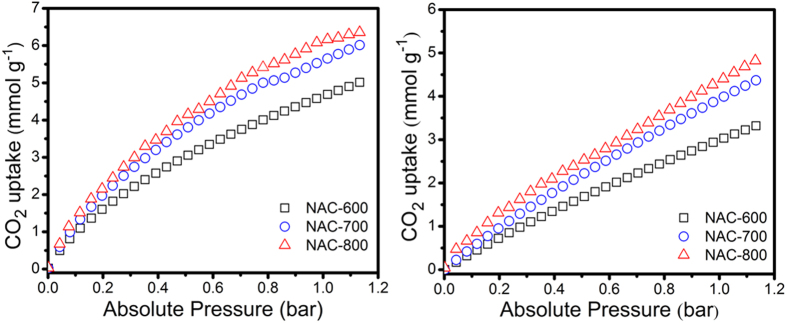
CO_2_ adsorption at 273 K (left) CO_2_ adsorption at 298 K (right).

The CO_2_ adsorption isotherms of NACs, measured by the volumetric method at 273 K, 298 K and 1.13 bar, are shown in Fig. 6. The presence of nitrogen-containing basic groups and large fraction of microporosity results in an increase in the synthesis temperature from 600 to 800 °C. All samples exhibit excellent CO_2_ capture capacities, which is regarded as one of the highest ever reported for porous carbon materials prepared from synthesized polymer. Figure 6 shows the CO_2_ adsorption isotherms of the activated carbon samples prepared under soft chemical activation conditions. Mildly activated carbons have high CO_2_ capture in the 5.01–6.35 mmol g^−1^ and 3.32–4.82 mmol g^−1^ ranges at 273 and 298 K respectively. NAC-800 shows that the highest CO_2_ capture is 6.35 mmol g^−1^. These data prove that specific surface area, tuneable pore size, optimum nitrogen content and chemical characteristics of the resulting porous activated carbon can be optimized in a systematic way to enhance the CO_2_ adsorption capacity. For comparison, the CO_2_ capture of original polymer (precursor) measured under the similar conditions shows a negligible adsorption, much less than 1.21 mmol g^−1^. Additionally, it is important to mention that the synthesized NAC-800 exhibits a larger adsorption capacity at low partial pressures (2.7 mmol g^−1^ at P/P_0_ = 0.2 and 273 K) than other recently synthesized materials. Noteworthy, for the NACs, the uptake of CO_2_ at a pressure of 0.2 bar is relatively high (2.7, 2.5 and 2.0 mmol g^−1^ at 273 K for NAC-800, NAC-700 and NAC-600 respectively). NAC-800 surpasses most porous carbons and is comparable with some high performing sorbents under the same or similar conditions (0.2 bar), such as NMC-800 (2.5 mmol g^−1^)^29^, PP1-2 (1.5 mmol g^−1^)^26^, AC (1.5 mmol g^−1^)^30^ and CS3-A6 (2.6 mmol g^−1^)^31^. It competes with the porous carbon materials and was comparable with some top performing CO_2_ adsorption such as NMC-800 (2.5 mmol g^−1^)^29^, NPC-650 (2.4 mmol g^−1^)^18^, MR-1-500 (2.2 mmol g^−1^)^30^. For this reason, we believe that this material has a potential to be applied as an industrial CO_2_ adsorbent for post combustion capture applications. However, further increment of the temperature cannot improve the CO_2_ adsorption.

### Heat of adsorption (Qst)

**Figure 7:**
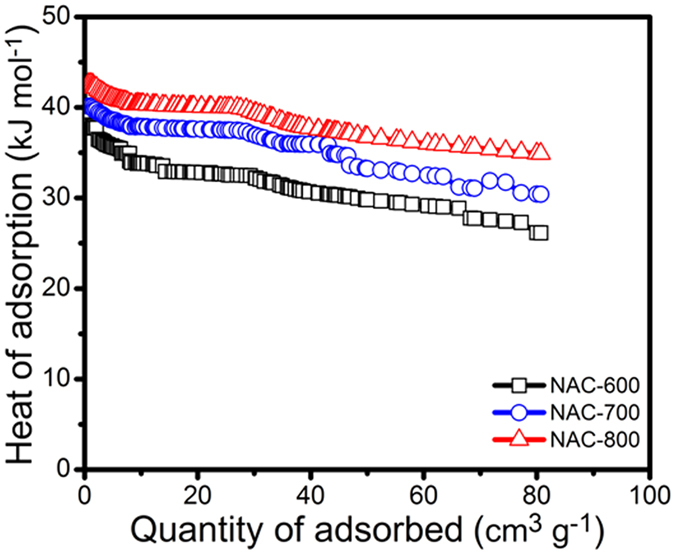
Heat of adsorption of CO_2_ for NACs calculated by the Clausius-Clapeyron equation.

To obtain more information about the interaction between the adsorbate and the adsorbent surfaces, the isosteric heat of adsorption (Qst) is derived from the CO_2_ adsorption isotherms at 273 K and 298 K. Qst (kJ mol^−1^) was calculated from the adsorption isotherms at different temperatures by using the Clausius−Clapeyron equation (the calculation process details see Supplementary information). The curves of Qst versus adsorption amount are shown in Fig. 7. As expected, at zero-loading, NAC-800 shows an heat of adsorption (Qst) of 42.6 kJ mol^−1^, comparatively higher than that of NAC-600 and NAC-700 (40.1 and 37.6 kJ mol^−1^) respectively. However, the Qst values are obviously bigger than those mentioned previously for typical carbonaceous adsorbents^18^,^29^,^32^. The high Qst value of NAC-800 is in consistent with the steep curve of the CO_2_. The relatively constant Qst indicates homogeneous binding sites over the full range of CO_2_ loading, which can be attributed to the high surface area, small pore size effect and the high polar framework. The dipole moment increases in the presence of N atoms on the AC surface and can facilitate the interaction between the NACs surface and CO_2_. Moreover, smaller pore size is also known to enhance the heat of adsorption.

### Selectivity

**Figure 8:**
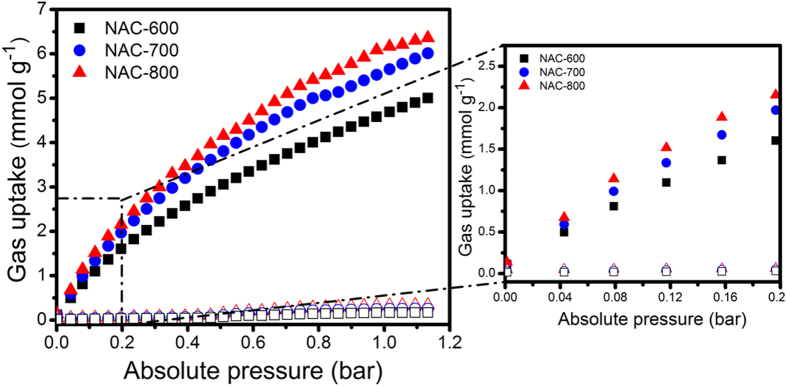
CO_2_ (solid) and N_2_ (open) adsorption isotherms of NACs at 273 K.

Recently, selectivity of CO_2_ over N_2_ is one of the most interesting features in the field of CO_2_ capturing materials. Selective adsorption behavior of these NACs for both gases (CO_2_ and N_2_) was also investigated using Henry’s law constants for single-component adsorption isotherms at 273 and 298 K and up to 1.13 bar (Fig. 8). In the low-pressure range (0.2 bar), the interaction between the NACs and CO_2_ play a dominant role for CO_2_ capture. Thus, the amount of CO_2_ adsorbed by NAC-800 (higher SSA and larger pore volume) is comparable to that of NAC-700 and NAC-600, indicating that NAC-800 has a greater affinity towards CO_2_. Among ACs, NAC-800 shows the highest CO_2_/N_2_ selectivity ratio of 123 at 273 K, whereas NAC-700 and NAC-600 exhibit ratios of 89 and 73, respectively. This comparatively large affinity of NAC-800 for CO_2_ may be attributed to the presence of basic nitrogen moieties and a large Qst. The optimal CO_2_/N_2_ selectivity value of NAC-800 at ambient temperature is even better than those of recently reported N-containing porous carbon materials^3^,^30^,^33^.

## Conclusions

In summary, the imine-linked polymers are shown to be good precursors for the preparation of porous carbon materials for CO_2_ capture and CO_2_/N_2_ selectivity. It is demonstrated that the nitrogen doping and porous structure of NACs favor the rapid trapping of CO_2_ at low pressure. Among NACs, NAC-800 has a greater affinity towards CO_2_ i.e., 6.35 mmol g^−^1 at 273 K and 4.82 mmol g^−1^ at 298 K and the highest CO_2_/N_2_ selectivity ratio of 123 at 273 K. More importantly, it has higher CO_2_ capture (2.70 mmol g^−1^ at 0.2 bar). These results clearly suggest that the NACs derived from polyimines are promising candidates for CO_2_ capturing and storage applications. The applicability of NACs can be further enhanced by increasing their specific surface area, optimization of the pore-size distribution and generating the nitrogen moieties on their surface.

## Method

### Synthesis of activated carbons

Activated porous carbon materials containing nitrogen (NACs) were prepared by direct pyrolysis of polyimine at different temperatures. Firstly, imine- containing monomer was synthesized following a previously reported procedure^18^. Through Schiff base reaction using aniline (2.8 g, 30 mmol) and benzaldehyde (3.18 g, 30 mmol) as the starting materials, dimethyl sulfoxide (DMSO, 120 mL) was added into a 250 mL round-bottom flask fitted with a water-cooled condenser and a magnetic stir bar. After being degassed by nitrogen, the mixture was heated at 180 °C overnight under magnetic stirring. It was then cooled to room temperature and the solid product was collected by filtration over a Büchner funnel and washed with a large amount of ethanol. The resulting yellow powder was then dried at 80 °C for 24 h.

Then, the imine-linked monomer (3.8 g, 20 mmol) dissolved in anhydrous dichloroethane (DCE, 20 mL), iron (III) chloride (6.69 g, 40 mmol) was then added. The resulting mixture was heated to 80 °C for 18 h. After cooling, the solid product was filtered and thoroughly washed with methanol until the filtrate turned clear. It was then further purified by soxhlet extraction in methanol for 24 h and dried in an oven vacuum at 60 °C for 24 h. Dark brown polymer was obtained in good quantitative yield.

After that, polyimine powders were directly carbonized in the tubular furnace under nitrogen (N_2_) atmosphere. The dry chemical activation of polyimine was carried out at 600–900 °C with KOH/polyimine weight ratio of 4:1. In a typical activation process, the mixture of polyimine with KOH pellets was put into a ceramic boat crucible in a horizontal tubular furnace. The mixture was heated to the target temperature for 1 h with the ramp temperature 10 °C/min under nitrogen gas flow. After cooling to room temperature, the obtained samples were thoroughly washed several times with 10 w% HCl to remove any inorganic salts, and large amount of distilled water until neutral pH was attained. Finally, the product was dried in a vacuum oven at 100 °C for 24 h. The obtained N-activated porous carbons were denoted as NAC-T, where NAC is the abbreviation of nitrogen doped activated carbons and T refers to the target temperature in °C. Note: NAC-900 was ignored because of the low surface area (423 m^2^ g^−1^) and low CO_2_ adsorption.

### Characterization of materials

The BET surface area (S_BET_), total pore volume and pore size distribution (PSD) of the samples were obtained from N_2_ adsorption/desorption isotherms at 77 K using an automatic Micromeritics ASAP 2020 surface area and porosity analyzer. Firstly, all samples were degassed at 110 °C for 8 h under vacuum (10^−5^ bar) prior to BET measurements. Part of the N_2_ adsorption isotherm used was of 99.999% purity (at 77 K and up to 1 bar) in the pressure (P/P_0_) range 0.04 to 0.2 fitted in the BET equation, and the BET surface area calculation was performed using all data points. The pore size distribution (PSD) was achieved from the nonlocal density functional theory (NLDFT) model in the Micromeritics ASAP2020 software package (assuming split pore geometry) based on the N_2_ sorption isotherm. In the gas sorption measurements, all gases used were of 99.999% purity. Low-pressure (0 to 1 bar), carbon dioxide (CO_2_ at 273 and 298 K) and nitrogen (N_2_ at 273 and 298 K) was used in sorption experiments carried out on a Micromeritics ASAP 2020 using an IGA-003 gravimetric adsorption instrument. Surface morphologies of the samples were examined by Field Emission Scanning Electron Microscopy (FE-SEM) (SIRION 200). High Resolution Transmission Electron Micrographs (HR-TEM) of carbon samples were collected by TECNAI G2 20 electron microscope operated at 200 kV. The components of activated carbon samples were investigated through elemental analysis to obtain % C, % H, % N and % O using Vario Micro cube Elemental Analyzer (Elementar, Germany). Fourier Transform Infrared (FT-IR) spectroscopy (Bruker Vertex 70 spectrometer) and X-ray photoelectron spectrometer (XPS -AXIS-ULTRA DLD high- performance imaging, Shimadzu, Japan) were used to analyze the functional groups on surface and the chemical composition of these materials.

## Additional Information

**How to cite this article**: Alabadi, A. *et al*. Imine-Linked Polymer Based Nitrogen-Doped Porous Activated Carbon for Efficient and Selective CO_2_ Capture. *Sci. Rep.* **6**, 38614; doi: 10.1038/srep38614 (2016).

**Publisher's note:** Springer Nature remains neutral with regard to jurisdictional claims in published maps and institutional affiliations.

## Supplementary Material

Supplementary Information

## Figures and Tables

**Table 1:** Texture Properties, Elemental Composite and XPS Analysis Data of NACs.

Sample	BET m^2^ g^−1^	Pore volume cm^3^ g^−1^	Element analysis			XPS analysis		
			C%	N%	O%	C%	N%	O%
NAC-600	1681	0.80	85.35	2.16	11.93	85.32	2.31	11.24
NAC-700	1719	0.85	88.72	2.07	9.81	87.11	2.02	10.18
NAC-800	1900	0.98	90.36	1.73	6.20	87.62	1.53	10.23
