# Supplementary material for: Imine-Linked Polymer Based Nitrogen-Doped Porous Activated Carbon for Efficient and Selective CO2 Capture
Source: Sci Rep. 2016 Dec 13;6:38614. doi: 10.1038/srep38614 (PMC5153834; doi:10.1038/srep38614)
Supplement: Supplementary Information [file srep38614-s1.pdf]

Supporting Information

Imine-Linked Polymer Based Nitrogen-Doped Porous Activated Carbon for Efficient and Selective CO_2 Capture

Akram Alabadi[1,2], Hayder A. Abbood[3], Qingyin Li[1], Ni Jing[1] and Bien Tan[1,]*

[1]School of Chemistry and Chemical Engineering, Huazhong University of Science and Technology, Luoyu Road No. 1037, Wuhan, 430074, China.

[2]South Refineries Company, Ministry of Oil, Basra, 61006, Iraq.

[3] Material Engineering Department, College of Engineering, University of Basrah, Basrah, 61006, Iraq.

*Correspondence to bien.tan@mail.hust.edu.cn

Heat of Adsorption Tabular Report

Quantity Adsorbed (cm^3/g STP)	Slope	Y-Intercept	Heat of Adsorption (kJ/mol)	Uncertainty (kJ/mol)
1.0000	-5202.1	20.968	43.253	0.000
2.4693	-5195.7	20.956	43.223	0.000
4.7767	-5063.3	20.750	43.154	0.000
6.1321	-4909.5	20.445	43.082	0.000
7.2040	-4900.9	20.427	43.048	0.000
9.2279	-4898.0	20.421	42.924	0.000
10.4874	-4777.9	20.188	42.903	0.000
10.9428	-4678.5	20.015	42.899	0.000
11.9386	-4670.3	19.998	42.831	0.000
13.1981	-4591.4	19.866	42.756	0.000
13.3419	-4581.7	19.848	42.634	0.000
14.5535	-4507.3	19.714	42.476	0.000
14.6733	-4500.7	19.703	42.220	0.000
15.9088	-4438.3	19.605	42.042	0.000
17.2642	-4368.7	19.483	41.923	0.000
17.4080	-4361.2	19.469	41.861	0.000
17.4559	-4358. 7	19.464	41.740	0.000
18.6195	-4298.5	19.351	41.647	0.000
19.9749	-4233.1	19.229	41.595	0.000
20.1426	-4225.1	19.214	41.429	0.000
21.3302	-4167.2	19.099	41.418	0.000
21.5698	-4155.4	19.075	41.349	0.000
22.6856	-4101.0	18.963	41.307	0.000
22.8773	-4091.9	18.944	41.282	0.000
24.0409	-4040.0	18.840	41.190	0.000
25.3963	-3984.5	18.732	41.029	0.000
25.6119	-3976.5	18.717	40.962	0.000
25.6838	-3973.9	18.712	40.841	0.000
26.7516	-3938.6	18.651	40.747	0.000
28.1070	-3901.3	18.597	40.637	0.000
28.3466	-3895.2	18.589	40.587	0.000
29.4623	-3868.0	18.553	40.681	0.000
29.7978	-3860.3	18.543	40.596	0.000
30.8177	-3837.7	18.516	40.408	0.000
31.0812	-3832.0	18.509	40.361	0.000
32.1730	-3807.9	18.478	40.251	0.000
33.5284	-3776.2	18.432	40.197	0.000
33.8159	-3769.1	18.420	40.038	0.000
33.9118	-3766.7	18.416	39.958	0.000
34.8837	-3740.9	18.371	39.903	0.000
36.2391	-3708.1	18.317	39.830	0.000
36.5506	-3701.4	18.307	39.775	0.000

36.9544	-3681.2	18.281	39.607	0.000
37.0257	-3673.5	18.272	39.543	0.000
37.9498	-3657.6	18.254	39.411	0.000
38.1852	-3652.1	18.248	39.365	0.000
38.3051	-3635.5	18.231	39.227	0.000
38.5199	-3606.0	18.194	39.182	0.000
38.7397	-3603.8	18.191	39.063	0.000
38.8545	-3560.5	18.136	38.983	0.000
39.2537	-3539.5	18.116	38.828	0.000
39.4892	-3522.4	18.098	38.786	0.000
39.6239	-3482.8	18.050	38.657	0.000
39.8676	-3480.7	18.048	38.540	0.000
39.9585	-3447.2	18.012	38.461	0.000
40.4816	-3428.9	17.995	38.309	0.000
40.6932	-3414.4	17.980	38.288	0.000
40.8278	-3385.3	17.958	38.147	0.000
40.9956	-3383.6	17.956	38.133	0.000
41.1625	-3357.6	17.936	37.916	0.000
42.1095	-3342.4	17.925	37.790	0.000
42.3971	-3330.3	17.913	37.690	0.000

The Langmuir adsorption isotherm assumes that adsorption takes place at specific homogeneous sites and is the simplest and still the most useful method. The Langmuir equation can be written as (1):

$$q = \frac{q_m \, b_p}{1 + b_p} \qquad (1)$$

Where q is the adsorbed quantity (mmol of CO_2 per g of NACs), p is the pressure of CO_2 in the bulk gas phase, q_m is the maximum adsorption capacity (mmol g^{-1}) and b is the Langmuir constant (bar^{-1}). Langmuir, isotherm model parameters and correlation coefficients for adsorption of CO_2 on NACs samples.

Samples	Langmuir parameters		R^2
	qm [mmol g^{-1}]	b [bar^{-1}]	
NAC-600	13.72	0.18	0.997
NAC-700	18.35	0.09	0.998
NAC-800	20.23	0.08	0.999

The Freundlich isotherm is an empirical equation used to describe heterogeneous systems. The Freundlich equation can be given by (2):

$$q = K_F \, P^{n-1} \qquad (2)$$

Where k_F is the Freundlich constant and n is the empirical constant associated with the adsorption driving force.

Samples	Freundlich parameters		R^2
	k_F [mmol g^{-1}]	1/n	
NAC-600	1.46	0.48	0.995
NAC-700	2.32	0.51	0.997
NAC-800	2.71	0.53	0.999
